# Rare and new cumaceans (Crustacea, Peracarida) from the southern margin of the Cap Ferret Canyon (Bay of Biscay)

**DOI:** 10.3897/zookeys.235.4027

**Published:** 2012-10-31

**Authors:** Jordi Corbera

**Affiliations:** 1 Carrer Gran, 90, E-08310 Argentona, Catalonia, Spain

**Keywords:** Cumacea, new species, deep sea, suprabenthos, Atlantic Ocean

## Abstract

A new cumacean genus and species, *Ithyleucon sorbei* **gen. et sp. n.**, was described from material collected in the southern margin of the Cap Ferret Canyon (Bay of Biscay, NE Atlantic). Although the new genus resembles *Pseudoleucon* Zimmer, 1903, in terms of the general aspect of the carapace and the pseudo-rostrum position, it shows important differences in the uropod structure and in the size of the antenna 1 accessory flagellum. In addition, some comments regarding the morphology of certain rare species (*Mesolamprops denticulatus* Ledoyer, 1983, *Hemilamprops normani* Bonnier, 1896 and *Schizocuma spino-culatum* (Jones, 1984)) are also provided.

## Introduction

Cumaceans display a wide diversity in deep waters (Jones and Sanders 1972) especially in low and mid latitudes (Gage et al. 2004). Within the Atlantic Ocean, the Bay of Biscay is probably the area best known for deep-sea cumacean fauna thanks to the works of Bonnier (1896), Fage (1929), Jones (1974, 1984, 1985), Reyss (1974a, 1978) and Bishop (1981a and b). However, despite the high sampling effort conducted in this geographical area, Elizalde et al. (1993) pointed out the presence of some rare and undescribed species during a study of suprabenthic communities of the southern margin of the Cap Ferret Canyon. Based in part on that material, Corbera et al. (in press) recently re-described *Campylaspis laevigata* Jones, 1974.

Following the study of suprabenthic communities of the Cap Ferret Canyon, this work deals with some rare and undescribed cumacean species that have since been discovered there.

## Material and methods

The present material was collected within the framework of a study on the suprabenthic community structure of the continental margin in the Bay of Biscay (Dauvin et al. 1985), During the ESSAIS I, ESSAIS II and ECOFER I surveys carried out between April and July 1989, 13 stations ranging from depths of 346 to 1099 m were sampled with a modified Macer-GIROQ suprabenthic sledge (full description in Dauvin et al. 1985). The collected material was fixed on board with a solution of 10% neutral formalin in seawater until subsequent sorting into major taxonomical groups at the laboratory. All groups (including cumaceans) were then transferred to 70% ethanol and so conserved until species identification. For morphological observations, the cumacean specimens were dissected in lactic acid and stained with chlorazol black. The dissected parts were mounted in Fauré medium and conserved in permanent glass slides sealed with nail varnish. Drawings were prepared using a camera lucida on an Olympus microscope.

The type material was deposited in the Biological Reference Collection (CBR) of the *Institut de Ciències del Mar*, CSIC, Barcelona.

## Taxonomy

### Family Lampropidae Sars, 1878

#### Mesolamprops denticulatus

Ledoyer, 1983

http://species-id.net/wiki/Mesolamprops_denticulatus

Fig. 1C

Mesolamprops denticulata – Ledoyer 1983, pp. 73–74, fig. 4; Ledoyer 1987, p. 68, fig. 5.
Mesolamprops sp. A – Elizalde et al. 1993, p. 250.
Mesolamprops denticulatus – Cartes et al. 2003, p. 749; Shalla and Bishop 2007, p. 1196.

##### Material examined.

Cap Ferret Canyon, Bay of Biscay, ESSAIS I: stn TS01, 44°33.30'N, 2°08.30'W, 346–347 m, 21/04/89, 2 pread. female. ESSAIS II: stn TS04, 44°34.380'N, 2°10.18'W, 484–485 m, 18/05/89, 1 pread. female. ECOFER I: stn TS05, 44°35.57'N, 2°11.21'W, 522–523 m, 1/07/89, 2 pread. female, 1 pread. male, 1 adult male. J.-C. Sorbe leg.

##### Remarks.

**Figure 1.**
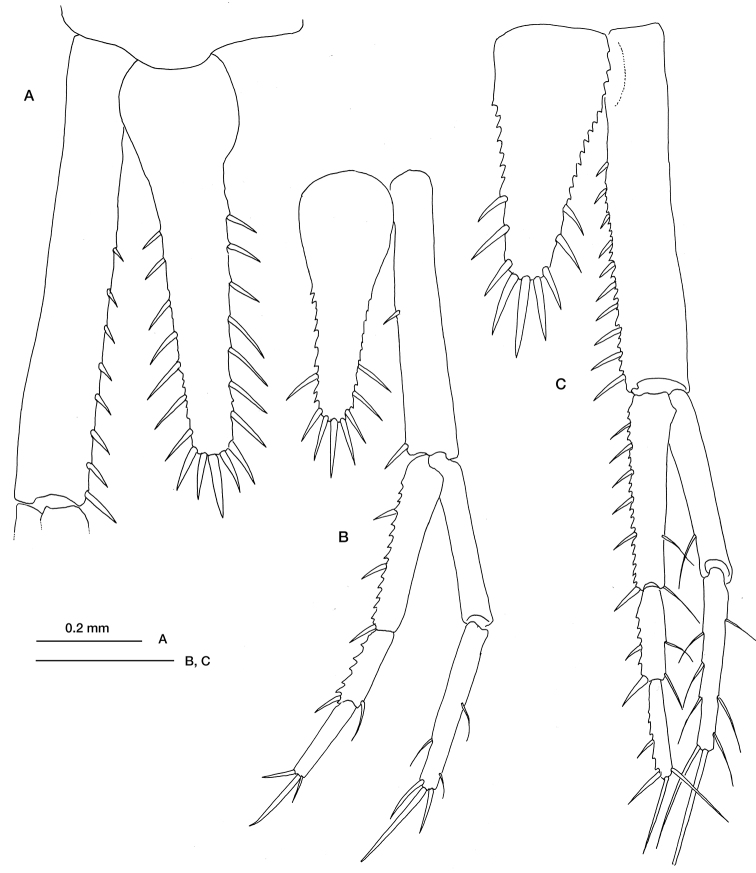
*Hemilamprops normani* Bonnier, 1896: **A** preadult female, telson and left uropod peduncle **B** manca, telson and right uropod. *Mesolamprops denticulatus* Ledoyer, 1983 **C** preadult female, telson and right uropod. Scale bar: 0.2 mm.

*Mesolamprops denticulatus* was described from the Mediterranean Sea by Ledoyer (1983), who identified the main diagnostic characteristics of the adult male (the flagellum of antenna 2 extending only to the end of thorax and two pairs of pleopods). Ledoyer also noted the difficulty of distinguishing the females of this species from those of two nearby species, *Hemilamprops normani* Bonnier, 1896 and *Hemilamprops cristatus* (Sars, 1870). Nevertheless, a detailed comparative study of the telson and uropod structures has allowed us to establish the main differences. In *Mesolamprops denticulatus* the telson has only 3–4 pairs of lateral setae (Fig. 1A); it is shorter than in *Hemilamprops*, and the terminal setae scarcely reach the distal end of the uropod peduncle; the central terminal seta is longer than the remaining two. Moreover, during the same developmental stage, *Mesolamprops denticulatus* remains smaller than *Hemilamprops normani*,based on measurements takenin preadult females (carapace length: 1.13 vs 1.93 mm).

##### Distribution.

*Mesolamprops denticulatus* was for a long time considered an endemic Mediterranean species until Shalla and Bishop (2007) reported the presence of this species in the Faeroe-Shetland Channel. In addition, Elizalde et al. (1993) recorded an undetermined *Mesolamprops* species from the Bay of Biscay, and recently a study of this same material confirmed that this specimens belongs to *Mesolamprops denticulatus* (Corbera and Sorbe in prep.). In the Mediterranean Sea, *Mesolamprops denticulatus* is distributed between depths of 170 and 570 m (Ledoyer 1983, 1987; Cartes et al. 2003), which is a bathymetric distribution pattern similar to that the observed in the Faeroe-Shetland Channel (259–753 m), as well as in the Bay of Biscay (346–708 m).

#### Hemilamprops normani

Bonnier, 1896

http://species-id.net/wiki/Hemilamprops_normani

Fig. 1A, B

Hemilamprops normani – Bonnier 1896, pp. 546–549, pl. 29 fig. 3.
Hemilamprops cristata – Calman 1905, p. 41, 49 [nec *Hemilamprops cristata* (Sars, 1870)].

##### Material examined. 

Cap Ferret Canyon, Bay of Biscay, ESSAIS II: stn TS10, 44°33.10'N, 2°13.13'W, 791–790 m, 18/05/89, 3 mancas, 2 pread. female, 1 ad. male; stn TS11, 44°32.89'N, 2°14.24’W, 923–924 m, 18/05/89, 6 mancas, 2 pread. males; stn TS13, 44°34.19'N, 2°16.18'W, 1097–1099 m, 17/05/89, 4 mancas, 2 imm. males. J.-C. Sorbe leg.

##### Remarks.

Although Calman (1905) suggested the synonymy between *Hemilamprops cristatus* and *Hemilamprops normani*, other authors (Sars 1900; Hansen 1920; Fage 1929, 1940) consider them as valid species, which is the criterion followed here. It is possible that the presence of *Mesolamprops denticulatus* in the Bay of Biscay, together with the two species of *Hemilamprops*, led to the confusion between these three species. *Hemilamprops normani* can be distinguished from the other two species by its higher number of lateral setae on the telson (6–8). Moreover, the three terminal setae of the telsonof *Hemilamprops cristatus* are of the same length, while in *Hemilamprops normani* the central one is the longest.

##### Distribution.

*Hemilamprops normani* is known to inhabit the waters of the Bay of Biscay (Bonnier 1896; Jones 1985), the west of Ireland (Calman 1905), the Azores Islands (Fage 1929) and the Mediterranean Sea (Fage 1940; Reyss 1974b). According to Jones (1985), in the Bay of Biscay this species inhabits bottoms between 280 and 3000 m. This wide bathymetric range, however, should not be assumed with complete certainty, since it is possible that *Hemilamprops normani* has been confused with *Hemilamprops denticulatus*, at least in its most shallow distribution. During this study *Hemilamprops normani* was always collected in waters deeper than 700 m.

### Family Leuconidae Sars, 1878

#### Ithyleucon gen. n.

urn:lsid:zoobank.org:act:D9D1A1D7-C6EA-4E26-9C19-7AE5DE138F31

http://species-id.net/wiki/Ithyleucon

##### Diagnosis.

Pseudorostrum extending anterodorsally and upturned; antenna 1 geniculate between peduncle article 1 and 2; accessory flagellum longer than main flagellum article 1; female with exopods on maxilliped 3 and pereopods 1–3; male with exopods on maxilliped 3 and pereopods 1–4; pereopod 2 ischium very short; uropod endopod 2-articulate; male with two pairs of pleopods.

##### Remarks.

The shape of the carapace and the position of the pseudorostrum of *Ithyleucon* gen. n. resemble those of *Pseudoleucon* Zimmer, 1903. However, *Ithyleucon* differs from the latter by 1) the size of the uropod endopod, which is longer than the peduncle and of similar length as the exopod (i.e., as long as the peduncle and certainly shorter than the exopod in *Pseudoleucon*) and by 2) the antenna 1 accessory flagellum, which is longer than the main flagellum article 1 (shorter in *Pseudoleucon*). Although these two features, as well as the geniculated antenna 1, are in agreement with the diagnosis of *Bytholeucon* Watling, 1991, the anterolateral corner is strongly angular in this genus and the known males observed up until now have had only one pair of pleopods.

In addition to these morphological differences, the only two known *Pseudoleucon* species also show divergence in terms of their ecology and biogeography. They inhabit shallow bottoms of the northeastern Pacific and a phylogenetic relationship with the genus described herein seems to be highly unlikely.

##### Etymology.

From the Greek *ithys*, meaning upright, referring to the position of the pseudorostrum, and *Leucon*, the stem genus. Gender masculine.

##### Type species.

*Ithyleucon sorbei* sp. n.

#### Ithyleucon sorbei sp. n.

urn:lsid:zoobank.org:act:B29AE99A-81B2-431D-B9DB-221C28671864

http://species-id.net/wiki/Ithyleucon_sorbei

Figs 2 [Fig F3] 4

Pseudoleucon sp. A – Elizalde et al. 1993, p. 253.

##### Material examined.

**Holotype:** Cap Ferret Canyon, Bay of Biscay, ESSAIS II, stn TS13, 44°34.19'N, 2°16.18'W, 1097–1099 m, 17/05/89, preadult female (ICMU12101901), Jean-Claude Sorbe leg.

**Paratypes:** Same data as the holotype, 1 preadult female (ICMU12101903), 1 preadult female dissected in two slides (ICMU12101902), 2 preadult males (ICMU12101904 and ICMU12101905); ESSAIS I, stn TS12, 44°32.30'N, 2°15.10'W, 1024–1043 m, 22/04/89 1 immature male (ICMU12101906), Jean-Claude Sorbe leg.

##### Diagnosis.

Carapace without ridges, frontal lobe with two teeth and others located posteriorly. Pseudorostral lobes extending anterodorsally, upturned, anterior margin serrate. Antenna 1 geniculate between peduncle articles 1 and 2, accessory flagellum extending beyond the mid-length of main flagellum. Female with exopods on pereopods 1–3; male with exopods on pereopods 1–4. Uropod peduncle shorter than rami; endopod bi-articulate, slightly shorter than exopod. Male with 2 pairs of pleopods.

##### Description.

**Figure 2.**
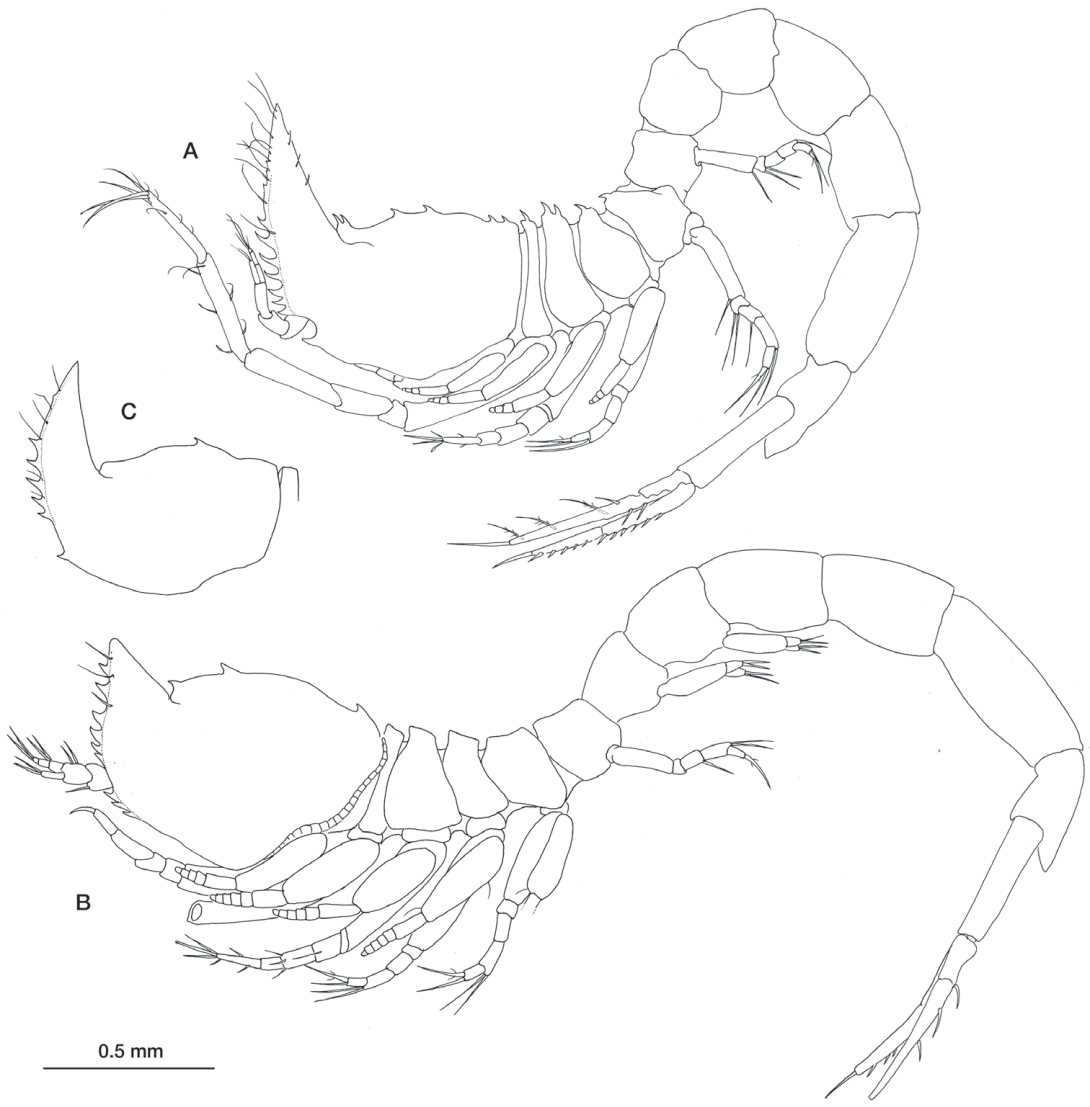
*Ithyleucon sorbei* gen. et sp. n. **A** preadult female holotype (ICMU12101901), whole animal in lateral view **B** preadult male paratype (ICMU12101904) **C** carapace of immature male paratype (ICMU12101906).

Preadult female 3.125 mm total length. Carapace (Fig. 2) slightly longer than a fourth of the total length; frontal lobe with two teeth and others (3–4) positioned posteriorly on the middorsal line. Pseudorostral lobes extending anterodorsally, upturned by an angle of about 90°, anterior margin serrate; antennal notch small, anterolateral angle acute with 0–3 serrations on the lower margin

**Figure 3. F3:**
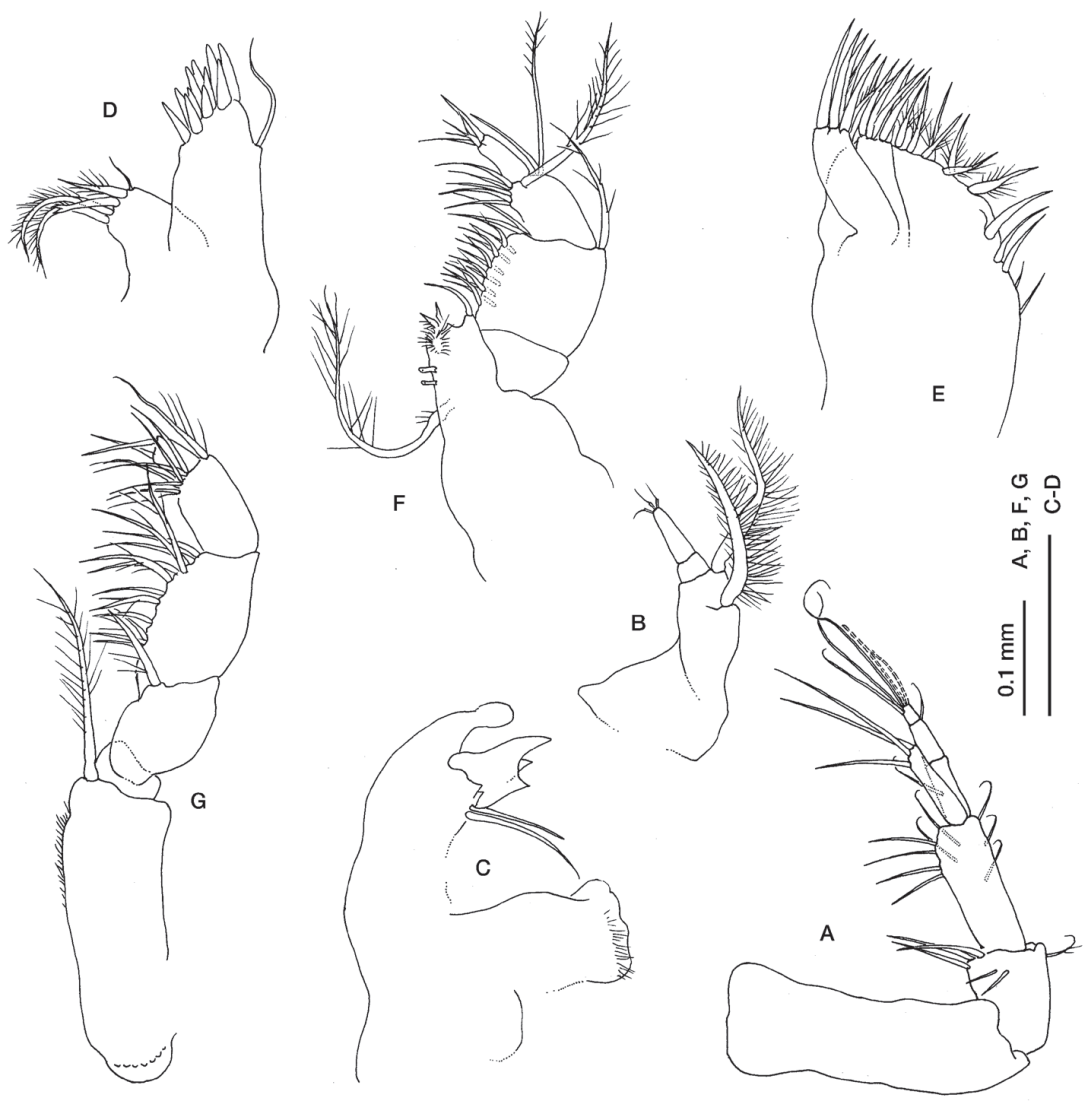
*Ithyleucon sorbei* gen. et sp. n. preadult female paratype (ICMU12101902): **A** antenna 1 **B** antenna 2 **C** left mandible **D** maxilla 1 **E** maxilla 2 **F** maxilliped 1 **G** maxilliped 2.

Antennula (Fig. 3A), peduncle 3-articulate, geniculate between articles 1 and 2; article 1 longer than the combined lengths of articles 2 and 3; article 2 shorter than article 3; main flagellum 3-articulate, shorter than the last peduncle article, with two aesthetascs and three long simple setae terminally; accessory flagellum longer than the main flagellum of article 1, with three long simple setae positioned terminally.

Antenna 2 (Fig. 3B) 3-articulate, with two pappose setae on article 1.

Mandible (Fig. 3C) base truncate, lacina mobilis with three teeth, two simple setae between lacina mobilis and pars molaris.

Maxillula (Fig. 3D) inner endite with five setae, one simple, three pappose and one bifid; outer endite with cuspidate setae.

Maxilla (Fig. 3E) with 3 endites; broad endite with 5 simple and several pappose setae terminally; narrow endites not extending beyond the distal margin of broad endite; inner narrow endite with 5 simple setae terminally; outer narrow endite with 4 simple setae terminally.

Maxilliped 1 (Fig. 3F) reduced with only three articles, dactylus minute.

Maxilliped 2 (Fig. 3G) basis shorter than rest of appendage, with a pappose seta on distal inner corner; merus with a long seta; carpus longer than merus with several simple setae on inner margin; propodus shorter than carpus, with a pappose seta on distal outer corner and several setae on inner margin; dactylus with two simple setae terminally.

**Figure 4.**
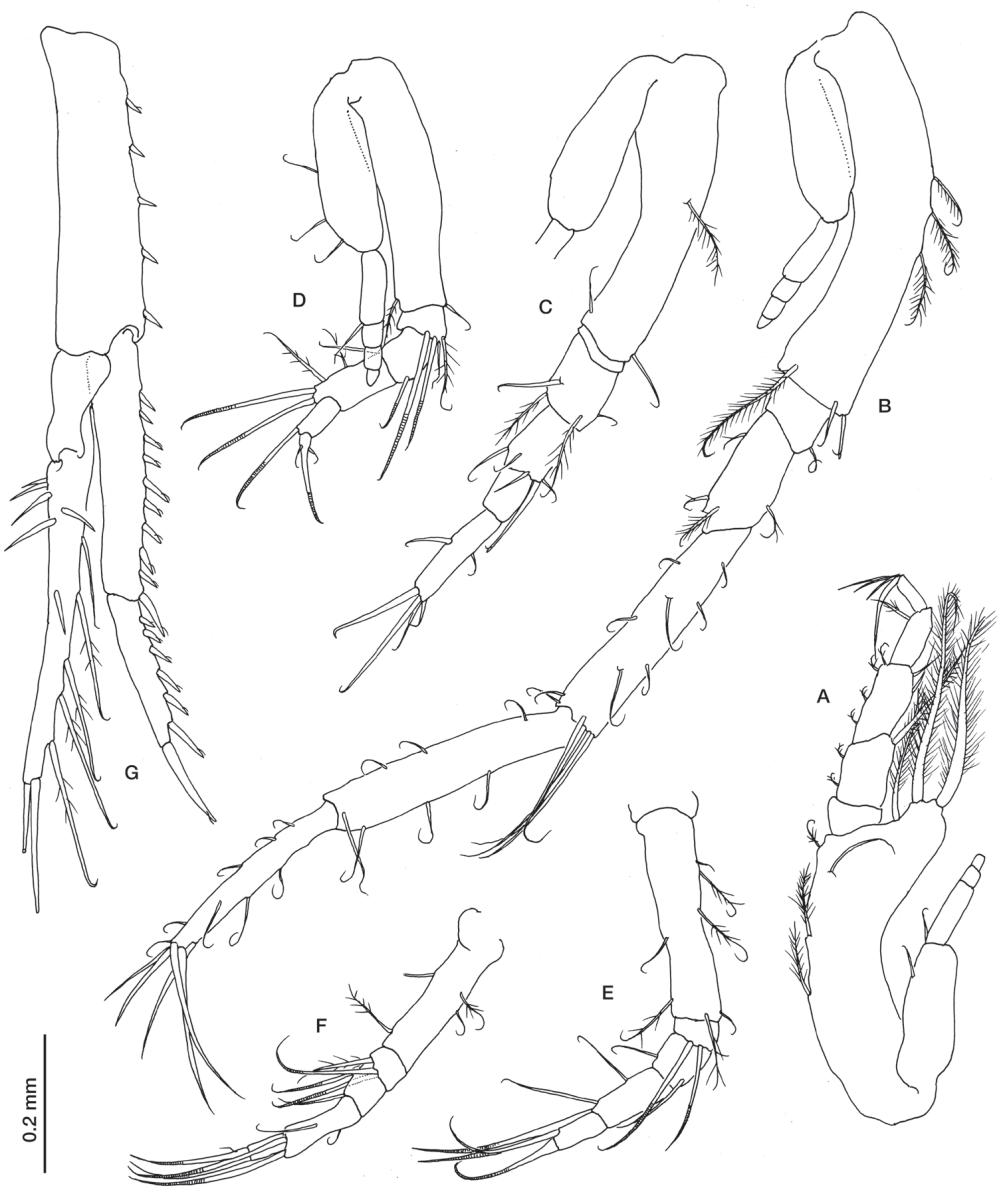
*Ithyleucon sorbei* gen. et sp. n. preadult female paratype (ICMU12101902): **A** maxilliped 3 **B** pereopod 1 **C** pereopod 2 **D** pereopod 3 **E** pereopod 4 **F** pereopod 5 **G** uropod.

Maxilliped 3 (Fig. 4A) with well developed exopod, basis longer than rest of appendage, produced distally, with three long pappose setae on distal outer corner and three pappose setae on inner margin; merus with small pappose sete on inner margin and a long pappose seta on distal outer corner; carpus as long as merus, with pappose seta on inner margin and two simple setae on distal outer corner; propodus shorter than carpus with a pappose seta on inner margin; dactylus shorter than propodus.

Pereopod 1 (Fig. 4B) with well developed exopod, basis shorter than the following three articles combined, with three pappose setae on its inner margin and a longer one on distal corner; ischium with a small simple seta on inner margin; merus half the length of carpus, with small pappose setae; carpus as long as propodus, with short simple setae on both margins and four long simple setae distally; propodus with simple setae on both margins; dactylus shorter than propodus, with five long simple setae terminally and some smaller ones along the margins.

Pereopod 2 (Fig. 4C) with well-developed exopod, basis as long as rest of appendage, with three pappose setae on inner margin and a long one on distal outer corner; ischium very short; as long as carpus; carpus with simple setae on distal margin; propodus half length of dactylus; dactylus with a simple setae on each margin and four terminally (the longest longer than the article).

Pereopod 3 (Fig. 4D) with well-developed exopod, basis longer than the rest of appendage, with a simple seta on distal anterior corner; ischium with three simple and a pappose setae on distal corner; merus twice as long as ischium, with a simple seta on distal corner; carpus twice as long as merus, with two long simple setae (distally annulated) on distal corner; propodus longer than half length of carpus with a long simple seta (distally annulated) on distal corner.

Pereopod 4 (Fig. 4E) basis as long as the rest of appendage, with simple and pappose setae on both margins; ischium with two long simple setae; merus with a simple seta on distal corner; carpus 1.5 times as long as merus, with two simple seta on the margin and two (distally annulated) on distal corner; propodus as long as merus, with a long simple seta (distally annulated) on distal corner.

Pereopod 5 (Fig. 4F), basis as long as the three following article combined length; carpus twice as long as merus, with two simple setae (distally annulated) on distal corner; propodus as long as merus, with a long simple seta (distally annulated) on distal corner.

Uropod peduncle (Fig. 4G) slightly longer than the last pleonite and 0.66 times as long as exopod, with five small cuspidate setae on inner margin. Endopod 2-articulate; article 1, 1.6 times as long as article 2, with 10 cuspidate setae on inner margin; article 2 with six cuspidate setae on inner margin and one terminally. Exopod 2-articulate, slightly longer than endopod; article 2 with simple setae on the outer margin and upper face, five pappose setae on inner margin, and two long simple setae terminally.

Preadult male 3.63 mm total length (Fig. 2B). Similar in most characteristics—apart from the sexual ones—to the female but with a shorter pseudorostrum, a lower number of teeth on the middorsal line and without antennal notch. However, the pseudorostrum of the immature male (pleopods reduced to a single bud with few terminal simple setae) is long as it is in females (Fig. 2C).

##### Etymology.

The new species is named in honour of Jean-Claude Sorbe (Arcachon, France) in recognition of his extensive work studying suprabenthic communities.

##### Distribution.

Bay of Biscay, N Atlantic between 1024 and 1099 m depth.

### Family Nannastacidae Bate, 1866

#### Schizocuma spinoculatum

(Jones, 1984)

http://species-id.net/wiki/Schizocuma_spinoculatum

Fig. 5

Cumella spinoculata – Jones 1984, pp. 219–220, fig. 10.
Schizocuma spinoculatum – Watling 1991, p. 755.

##### Material examined.

*Schizocuma spinoculatum*: ESSAIS II, stn TS13, 44°34.19'N, 2°16.18'W, 1097–1099 m, 17/05/89, 7 pread. females, 2 imm. males, 1 ad. male.

*Schizocuma molosa* (Zimmer, 1907): BENTART 06; stn 30, 69°58'24"S, 87°26'54"W, 1798–1799 m, 27/01/2006, 1 ad. male, 1 imm; stn 31, 69°57'46"S, 87°22'08"W, 1395 m, 29/01/2006, 2 imm. females, 1 ad. male; stn 38, 69°15'11"S, 80°12'11"W, 1339–1343 m, 5/02/2006, 1 imm. female.

##### Remarks.

**Figure 5.**
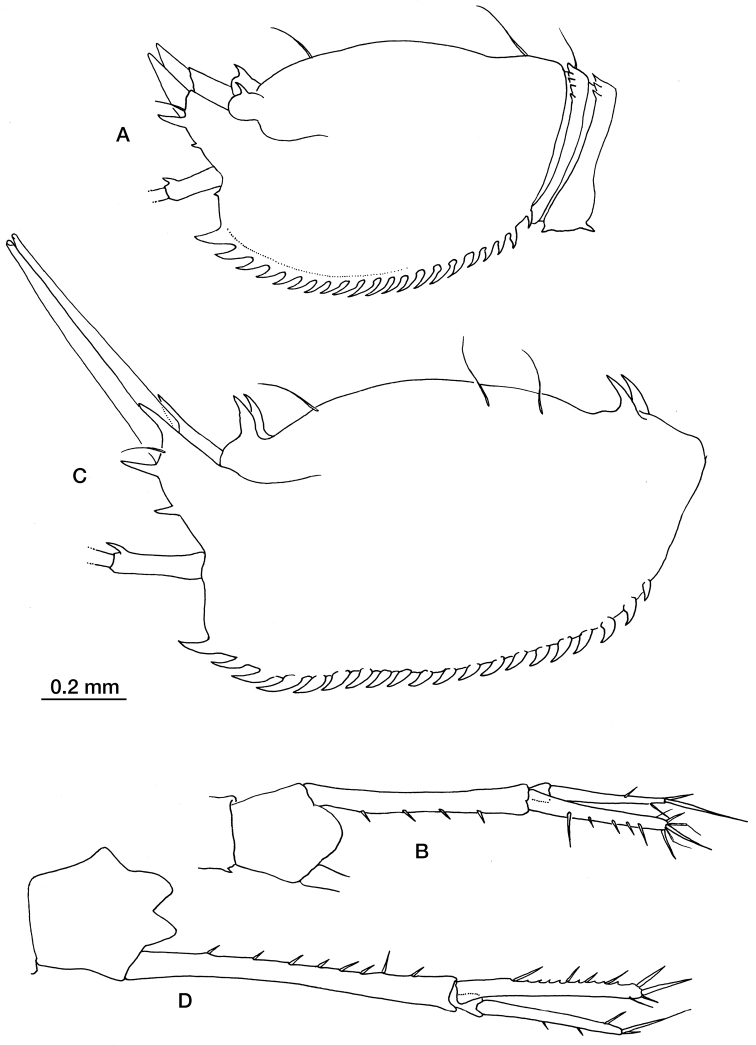
*Schizocuma spinoculatum* (Jones, 1984): **A** carapace in lateral view **B** uropod. *Schizocuma molosa* (Zimmer, 1907) from Bellingshausen Sea, Antarctica: **C** carapace in lateral view **D** uropod.

When Jones (1984) described *Schizocuma spinoculatum*, he had already noted its strong resemblance to *Schizocuma molosa*, but then the latter species was only known by a single partially broken specimen (Zimmer 1907, 1913). Comparison of the material collected in the Bay of Biscay with those obtained during the Bentart 06 cruise in the Bellingshausen Sea (Corbera et al. 2009) enabled us to identify differences between the two species. On the tip of pseudorostrum of *Schizocuma molosa* there are a couple of spines (Fig. 5C), one on the upper angle and another just bellow the siphon; the first one is absent in *Schizocuma spinoculatum* (Fig. 5A). The hinder dorsal third of the carapace of *Schizocuma molosa* also has a pair of forward curving spines and long simple setae while *Schizocuma spinoculatum* has only a pair of simple setae. Moreover, the carapace is more elongated in *Schizocuma molosa* than in *Schizocuma spinoculata* (length-height ratio: 1.8 vs 1.5), which is also true of the uropod peduncle (peduncle-endopod length ratio: 1.8–1.9 vs 1.5) (Fig. 5B, D). All of these differences can be observed both in males and in females, which gives support to the validity of both species.

## Supplementary Material

XML Treatment for Mesolamprops denticulatus

XML Treatment for Hemilamprops normani

XML Treatment for Ithyleucon

XML Treatment for Ithyleucon sorbei

XML Treatment for Schizocuma spinoculatum

## References

[B1] Bonnier JJ (1896) Édriophthalmes. Résultats Scientifiques de la Campagne du “Caudan” dans le Golfe de Goscogne, Août-Septembre 1895. Annales de l’Universitée de Lyon 26 (3): 528-562.

[B2] Calman WT (1905) The marine fauna of the west coast of Ireland. Part IV. Cumacea. Fishery Ireland Scientific Investigations 1: 1-52.

[B3] Bishop JDD (1981a) Two new leuconids (Peracarida, Cumacea) of widespread occurrence in the deep Atlantic. Crustaceana 40 (2): 144-159. doi: 10.1163/156854081X00552

[B4] Bishop JDD (1981b) A revised definition of the genus *Epileucon* Jones (Crustacea, Cumacea), with descriptions of species from the deep Atlantic. Philosophical Transactions of the Royal Society of London. Series B. Biological Sciences 291 (1052): 353-409. doi: 10.1098/rstb.1981.0003

[B5] Cartes J Jaume D Madurell T (2003) Local changes in the composition and community structure of suprabenthic peracarid crustaceans on the bathyal Mediterranean: influence of environmental factors. Marine Biology 143 (4): 745-758. doi: 10.1007/s00227-003-1090-z

[B6] Corbera J San Vicente C Sorbe J (2009) Cumaceans (Crustacea) from the Bellingshausen Sea and off the western Antarctic Peninsula: a deep-water link with fauna of the surrounding oceans. Polar Biology 32 (4): 611-622. doi: 10.1007/s00300-008-0561-6

[B7] Corbera J Galil BS Sorbe J (in press) First record of *Campylaspis laevigata* (Crustacea: Cumacea: Nannastacidae) in the Mediterranean Sea: redescription and ecological notes. Marine Biodiversity Records.

[B8] Dauvin JC Sorbe JC (1995) Suprabenthic amphipods from the southern margin of the Cap-Ferret canyon (Bay of Biscay, Northeastern Atlantic Ocean): abundance and bathymetric distribution. Polskie Archiwum Hydrobiologii 42: 441-460.

[B9] Dauvin JC Sorbe JC Lorgeré JC (1985) Benthic Boundary Layer macrofauna from the upper continental slope and the Cap Ferret canyon (Bay of Biscay). Oceanologica Acta 18: 113-122.

[B10] Elizalde M Sorbe JC Dauvin JC (1993) Las comunidades suprabentónicas batiales del golfo de Vizcaya (margen sur del cañón de Cap-Ferret): composición faunística y estructura. Publicaciones Especiales del Instituto Español de Oceanografía 11: 247-258.

[B11] Fage L (1929) Cumacés et Leptostracés provenants des campagnes scientifiques du Prince Albert 1er de Monaco. Resultats des Campagnes Scientifiques 77: 1-50.

[B12] Fage L (1940) Les Cumacés de la Méditerranée. Remarques systematiques et biologiques. Bulletin de l’Institute oceanographique, Monaco 783: 1-14.

[B13] Gage JD Lambshead PJD Bishop JDD Stuart CT Jones NS (2004) Large-scale biodiversity pattern of cumacea (Peracarida: Crustacea) in the deep Atlantic. Marine Ecology Progress Series 277: 181-196. doi: 10.3354/meps277181

[B14] Hansen H (1920) Cumacea. Crustacea Malacostraca 4. The Danish Ingolf-Expedition 3 (6): 1-86.

[B15] Jones NS (1974) *Campylaspis* species (Crustacea: Cumacea) from the deep Atlantic. Bulletin of the British Museum (Natural History), Zoology 27: 247-300.

[B16] Jones NS (1984) The family Nannastacidae (Crustacea: Cumacea) from the deep Atlantic. Bulletin of the British Museum (Natural History), Zoology 46: 207-289.

[B17] Jones NS (1985) Distribution of the Cumacea. In: Laubier L Monniot C (Eds) Peuplements profonds du golfe de Gascogne: campagnes BIOGAS, IFREMER, Brest. 429–433.

[B18] Jones N Sanders H (1972) Distribution of cumacea in the deep atlantic. Deep Sea Research 19: 737-745.

[B19] Ledoyer M (1983) Contribution à l’étude de l’écologie de la faune vagile profonde de la Méditerranée nord-occidentale. II: Les cumacés (Crustacea). Tethys 11 (1): 67-81.

[B20] Ledoyer M (1987) Les Cumaces mediterranees profonds (Crustacea) des campagnes Biomedes I et II et Balgim. Synthese de la distribution bathyale du groupe en Mediterranee occidentale. Mesogee 47: 59-70.

[B21] Reyss D (1974a) Contribution a l’étude des cumacés de profondeur de l’Atlantique nord: le genre *Makrokylindrus* Stebbing. Crustaceana 26: 5-28. doi: 10.1163/156854074X00028

[B22] Reyss D (1974b) Cumacés Résultats scientifiques de la campagne “Polymède II” du NO “Jean Charcot” en Mer Ionienne et en Mer Égée (Avril-Mai 1972). Crustaceana 27 (2): 216-222. doi: 10.1163/156854074X00442

[B23] Reyss D (1978) Cumacés de profondeur de l’Atlantique nord. Famille des Lampropidae. Crustaceana 35: 1-21. doi: 10.1163/156854078X00150

[B24] Sars GO (1900) An acount of the Crustacea of Norway. Vol. 3. Cumacea. Bergen Museum, Christiania, Oslo, 115 pp.

[B25] Shalla SH Bishop JDD (2007) Lampropidae (Crustacea: Cumacea) from the deep north-east Atlantic and the North Sea, with two new species of Hemilamprops and Mesolamprops. Journal of the Marine Biological Association of the United Kingdom 87 (5): 1191-1200. doi: 10.1017/S0025315407055063

[B26] Watling L (1991) Rediagnosis and revision of some Nannastacidae (Crustacea: Cumacea). Proceedings of the Biological Society of Washington 104 (4): 751-757.

[B27] Zimmer C (1907) Neue Cumacenn von der Deutschen und Schwedischen Südpolar-Expedition aus Familien der Cumiden, Vaunthompsoniiden, Nannastaciden und Lampropiden. Zoologischer Anzeiger 31: 367-374.

[B28] Zimmer C (1913) Die Cumaceen der Deutchen Südpolar-Expedition 1901–03. Deutsche Südpolar-Expedition (1901–1903) 14(6): 437–491.

